# Climbing as an Add-On Treatment Option for Patients with Severe Anxiety Disorders and PTSD: Feasibility Analysis and First Results of a Randomized Controlled Longitudinal Clinical Pilot Trial

**DOI:** 10.3390/ijerph191811622

**Published:** 2022-09-15

**Authors:** Carina S. Bichler, Martin Niedermeier, Katharina Hüfner, Mátyás Gálffy, Johanna M. Gostner, Philipp Nelles, Stefanie E. Schöttl, Barbara Sperner-Unterweger, Martin Kopp

**Affiliations:** 1 Department of Sport Science, University of Innsbruck, Fuerstenweg 185, 6020 Innsbruck, Austria; 2 Department of Psychiatry, Psychotherapy, Psychosomatics and Medical Psychology, University Hospital of Psychiatry II, Innsbruck Medical University, Anichstraße 35, 6020 Innsbruck, Austria; 3 Institute of Medical Biochemistry, Biocenter, Medical University of Innsbruck, Innrain 80-82, 6020 Innsbruck, Austria

**Keywords:** anxiety disorder, post-traumatic stress disorder, exercise, physical activity, climbing therapy, biomarker

## Abstract

Background: Exercise has considerable effects on physical and psychological health. Anxiolytic effects of climbing exercise have been found in people suffering from depression. However, there are no studies on patients with severe anxiety disorders or post-traumatic stress disorder (PTSD) practicing climbing as add-on treatment. Additionally, many studies on physical therapy fail to use adequate active control groups. Therefore, this study aimed to investigate the feasibility of a four-week climbing exercise program for patients with anxiety disorders or PTSD in comparison to a standard exercise treatment and a social control group. Methods: Outpatients diagnosed with anxiety disorders or PTSD (F 40, F 41, F 43.1 according to ICD-10) were randomly assigned to (a) climbing exercise (n = 27), (b) Nordic walking exercise (n = 23), or (c) control condition (n = 23) providing the same amount of social contact for eight sessions of 90 minutes each. Psychological parameters (symptom severity, worry symptoms, self-efficacy, quality of life) and biological parameters were assessed at the beginning and at the end of the four-week program. Additionally, follow-up assessments were conducted three and six months after the program ended. Results: Sixty outpatients (75% female) aged 18–65 years with a longstanding history of a mental disorder (>10 years) and classified as treatment-resistant (95%) and with averaging 3.8 psychiatric comorbidities completed the pilot trial. After participation, symptoms of anxiety disorders were significantly reduced (*p* = 0.003), and health-related characteristics significantly improved (depression symptoms: *p* < 0.001, worry symptoms: *p* < 0.001, self-efficacy: *p* < 0.001, quality of life-physical health: *p* = 0.002, quality of life-psychological health: *p* = 0.006) in all groups. The feasibility of conducting climbing exercises for the patient groups could be demonstrated, and a general acceptance in the groups was recorded. No significant time-by-group interactions were found. At the completion of the program, psychological parameters improved, while biological parameters remained the same in all three groups. Conclusions: Participation in the climbing group as well as in Nordic walking and social contact groups demonstrated beneficial results in patients with anxiety disorders and PTSD with severe mental burden. Nevertheless, climbing did not show any additional clinically relevant benefits compared to Nordic walking or social contact. Studies with larger sample sizes and qualitative insights are needed to further evaluate the possible benefits of climbing in this population.

## 1. Introduction

Anxiety disorders, trauma, and stress-related disorders such as post-traumatic stress disorder (PTSD) are potentially chronic, disabling conditions that are distributed across the globe [1]. These disorders are the sixth leading cause of years lived with disability, regardless of the income in the countries (from low- to high-income countries) [1]. Among different types of anxiety disorders and PTSD, the estimated worldwide lifetime prevalence ranges between four and 25% in the general population [2,3].

Anxiety disorders and PTSD are associated with several negative physiological and psychological impairments such as increased risk of cardiovascular disease and mortality [4] and reduced quality of life [5]. Most people with anxiety disorders and PTSD do not seek treatment, possibly due to stigmatization [6]. Of those who seek treatment, one-third do not respond to frontline treatments like psychotherapy (e.g., cognitive behavioral therapy) and pharmacological agents (e.g., SSRIs) [7]. If a treatment attempt with at least one antidepressant medication in adequate dosage and duration and cognitive behavioral therapy remain unsuccessful, the disorder is said to be treatment-resistant or refractory anxiety disorder/PTSD [8,9]. Exercise-based interventions might be an alternative add-on treatment option for:

1. Individuals who are non-responders or treatment-resistant;
2. Individuals from regions where evidence-based interventions are inaccessible or not acceptable (e.g., stigmatization);
3. Individuals who have already developed or are at risk of developing physical health problems (e.g., cardiovascular disease and high blood pressure) that are associated with anxiety disorders [7,10].

Several mechanisms of physical activity in general and exercise in particular that may reduce anxiety disorder and PTSD symptoms have been explored in the area of exercise medicine: exposure and desensitization to internal arousal cues, enhanced cognitive function, exercise-induced neuroplasticity, normalization of hypothalamic–pituitary axis function, and reductions in inflammatory markers [11]. In addition, physical activity is also a protective factor against the development of an anxiety disorder and PTSD [12]. 

A systematic review and meta-analysis [7] as well as its update [13] summarize that exercise significantly decreases anxiety symptoms more than control conditions do in people with anxiety or stress-related disorders with a moderate effect size, equal to the anxiolytic effects from common pharmacotherapy. Similar exercise effects were reported also for individuals living with PTSD [14,15]. These results suggest that exercise can be effective in decreasing anxiety symptoms for patients with a current diagnosis of anxiety or stress-related disorders (e.g., PTSD). 

On the other hand, a meta-analysis [16] reported no effectiveness of aerobic exercise in the treatment of anxiety disorders. Even for individuals living with depressive disorders, where more evidence is available, the positive effect of physical activity is frequently but not uniformly present [17].

Additionally, most reviews request more research to answer open questions, e.g., the modality of exercise that is most effective in treatment. Choosing an appropriate exercise program is a challenge for many facilities providing treatment for people with anxiety disorders or PTSD. While some do not provide any exercise program at all, others often offer Nordic walking as standard exercise. Nordic walking is a walking technique with poles and has been shown in previous studies to be a suitable exercise program, especially due to its ease of implementation and high preference in patients [18]. Nevertheless, other exercise programs should also undergo scientific review, even if initially they may be more difficult to implement. In addition to yoga and resistance-based trainings (e.g., [14], climbing and bouldering (rock climbing to moderate heights without rope) have recently been shown to be promising exercise interventions [19,20,21,22].

Climbing exercise is a form of resistance training with potential positive influences on physiological and mental health in the general population and in individuals with psychiatric conditions in particular [23,24]. Previous studies reported, among other benefits on a short- and long-term level [25,26], symptom reduction [19,20,21,27], increased self-efficacy [28], and reduced trait anxiety [29] in patients with depressive disorders. A recently published study showed similar effects of cognitive behavioral therapy and boulder psychotherapy, in a sample of depressed patients predominantly in their first or second depressive episode, mostly without comorbidities [22].

The efficacy of climbing therapy in different patient groups needs to be further assessed [27]. While climbing exercise may have great potential for depressive patients, the available literature in patients with anxiety disorders and PTSD is scarce. Climbing exercise could be helpful for this group of patients due to its appealing character and acceptability, socially interactive aspects (e.g., mutual trust in belaying), and the numerous controllable exposure tasks [22]. A major problem is the implementation of exercise programs for patients, especially for severely affected individuals. For exercise studies of patients with anxiety and stress-related disorders, drop-out rates of 22% were observed, and a high publication bias must be assumed in this research field [30].

As exercise interventions are often performed in groups, it is possible that social contact/support promotes health-related symptom reduction. The reduction of social withdrawal [30] and the increase of social interaction is one of the mechanisms seen responsible for the anxiolytic effect of physical activity [31]. From a biological perspective, it has not yet been clarified how exercise affects biological parameters. In this context, the content of neurotransmitters is of interest, whose availability depends in particular on the turnover of precursor molecules such as the amino acids tryptophan and phenylalanine. Both phenylalanine and tryptophan are essential amino acids, providing a biochemical link between nutrition, oxidative stress and psychobiochemistry [32]. Tryptophan is the precursor in the synthesis of serotonin, while phenylalanine is crucial for dopamine, noradrenaline, and adrenaline formation [33]. These neurotransmitters are the main targets in the treatment of various psychiatric disorders such as depression, anxiety, and other mood disorders [34]. Moreover, the complex interplay between immunological processes and the previously mentioned pathways must be considered [35]. Therefore, intervention studies on physical activity are needed to clarify the simultaneous but complex effects on neurotransmitter precursor amino acid pathways and changes in psychological parameters [36].

To answer these clinically relevant questions, controlled longitudinal trials are needed to compare different group exercise strategies and its additional exercise effects [24].

Therefore, the current study aims to:

(1) Assess the acceptance and feasibility of climbing therapy for patients diagnosed with anxiety disorders and PTSD, including patients with severe mental burden.
(2) Compare the efficacy on health-related aspects, such as symptom severity, between climbing, Nordic walking, and non-exercise social contact control programs.
(3) Assess the possibility of simultaneously collecting biological parameters to identify underlying pathobiochemical mechanisms.

## 2. Materials and Method

### 2.1. Study Design and Procedures

This was a longitudinal controlled clinical pilot trial with random allocation of the participants to one of three groups: climbing, Nordic walking, social contact control. Participation in the groups was offered as an add-on to a regular, ongoing clinical outpatient care program consisting of psychological, psychotherapeutic, and psychopharmacological therapy. Additionally in this study, affective reactions to each individual program session were collected, and results have been reported previously [26].

Participants were screened in the Department of Psychiatry, Psychotherapy, Psychosomatics and Medical Psychology at the Medical University of Innsbruck. When assessed as eligible for the study, outpatients were invited to an informative meeting and randomly allocated to one of three groups by use of a computer-generated, blockwise cluster-randomization scheme with an allocation ratio of 1:1:1. Block sizes were a minimum of three and a maximum of eight. Whether participants had a diagnosis of an anxiety disorder vs. PTSD was not considered in randomization. This procedure was of an organizational nature so that the participants were offered the earliest possible start of the study program to reduce drop out.

**Figure 1:**
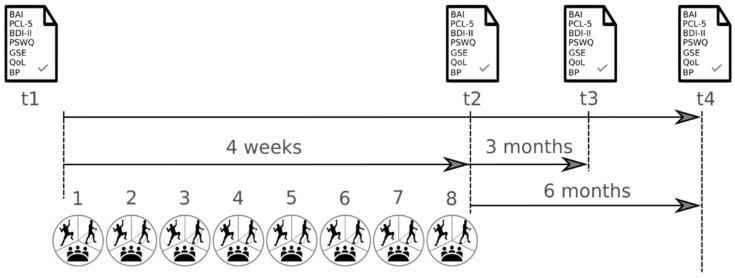
Schedule of the project. Measurement of study outcomes at four timepoints. Notes: BAI: Beck Anxiety Inventory, PCL-5: Post-traumatic Stress Disorder Checklist, BDI-II: Beck Depression Inventory, PSWQ: Penn State Worry Questionnaire, GSE: General Self-Efficacy, QoL: Quality of Life, BP: biological parameters, t1: before the program, t2: at the end of the program, t3: three-month follow-up, t4: six-month follow-up, ✓: completed questionnaires, 1–8: attended sessions.

Participants received study information and timetables for their group attendances. After giving written informed consent, the program started within two weeks and the period ended after eight group sessions within four weeks. Study outcomes were assessed before the program (baseline, t1), post-program (t2), at three months post-program (t3), and at six months post-program (t4) (see Figure 1). Ethics Committee endorsement for the trial was obtained from the Medical University of Innsbruck: 1139/2017. Furthermore, the trial was registered at http://www.clinicaltrials.gov/ on 29 November 2018 with the indication code NCT03758599.

### 2.2. Participants

The participants were recruited by local treatment staff. 

Inclusion criteria were:

(a) outpatients with a primary ICD-10 diagnosis based on a clinical judgement of any anxiety disorder or post-traumatic stress disorder (PTSD) (F 40, F 41, F 43.1; ICD-10; World Health Organization),
(b) aged between 18–65 years, and
(c) giving written informed consent.

Exclusion criteria were:

(a) patients with acute psychosis or suicidal behavior,
(b) medical contraindication to exercise assessed by a clinician,
(c) somatic comorbidity with contraindication to moderate physical activity (e.g., high risk of cardiac events; judged by the patients’ primary care physician),
(d) cognitive deficits (unsuitable to complete the required questionnaires, diagnosed by the referring psychiatrist, psychologist, or psychotherapist),
(e) deficiencies in German language skills (failing to complete the required questionnaires, verbally interact in the social contact group, or understand the instructions given in the exercise groups; this was diagnosed by the referring psychiatrist, psychologist, or psychotherapist).

Sociodemographic, clinical, and health-related data were gathered from the patients prior to the program as well as from medical charts. Medical charts were accessed from the computerized documentation system of the Department of Psychiatry, Psychotherapy, Psychosomatics and Medical Psychology Innsbruck. Data included medication status, information of the diagnosis, and hospital visits for inpatient treatment and outpatient consultations in the acute care hospital at the Department of Psychiatry, Psychotherapy, Psychosomatics and Medical Psychology at the Medical University of Innsbruck. No medical records from other treatment facilities or registered practitioners were consulted. Further, the years of treatment in the Department of Psychiatry, Psychotherapy, Psychosomatics and Medical Psychology at the Medical University of Innsbruck were reported. Health-related data included physical activity status, body mass index, blood pressure, and smoking status. Patients were monitored throughout the study. Changes in medication, psychological, and psychotherapeutic treatment, as well as any other exceptional life events were noted and recorded throughout follow-up treatment.

The sample size was calculated a priori using power analysis G*Power with the following assumptions: The effect size of exercise in reducing symptoms of anxiety was assumed to be a standardized mean difference of 0.58 (equaling an effect size of f = 0.29; [37]) based on a review exclusively focusing on studies using an intent-to treat approach [7]. A total sample size of n = 165 was calculated to detect an effect size of f = 0.29 in the group by time interaction of a three × four mixed analyses of variances (α = 0.05, β = 0.20, non-sphericity correction = 1, and options: as in [37]). Since reaching a sample size of 165 patients was not possible in this single-center setting within the timescale interrupted by the COVID-19 pandemic, the study was closed, and the data were published as a pilot trial.

### 2.3. Programs

All programs were led by two professionals. These varied across different disciplines, including clinical health psychology, sports and exercise psychology, sport science, training therapy, and medicine. All programs consisted of eight 90-minute sessions spread over four weeks (two sessions per week). Both exercise programs were intentionally designed as exercises rather than conversation sessions. In case the exercise situations brought up anxiety and PTSD-related topics, these could directly be discussed with the professionals. Both exercise programs were performed with comparable intensity. Participants in both exercise programs were instructed to perform the overall impression of the sessions with moderate intensity based on the subjective rating of perceived exertion that was introduced [38].

#### 2.3.1. Climbing Exercise

Each session began with a standardized body-centered warm-up of ten minutes identical to that in the Nordic walking group. The group sessions took place outdoors if the weather and temperature conditions allowed. In bad weather conditions, the indoor area was used. The general warm-up was followed by a climbing-specific warm-up, which consisted of bouldering (i.e., climbing horizontally without the use of a rope near the floor) for around 25 min. After warm-ups, the actual rope climbing sessions lasted around 45 min. During the first five sessions of rope climbing, only the therapist provided rope security. Safety and belaying of partners were demonstrated and explained throughout those sessions. Depending on participants’ knowledge and skills, they were allowed to belay each other; however, always guided by a therapist. Climbing sessions contained sport-specific training to familiarize the participants with gear and rope management, to learn footwork and route finding, and to locate good belay spots and resting positions while climbing. Every climbing session ended with a short cool-down session of five minutes (i.e., stretching) identical to that in the Nordic walking group.

#### 2.3.2. Nordic Walking Exercise

The Nordic walking group started with a ten-minute body-centered warm-up identical to that in the climbing group, followed by a five-minute Nordic-walking-specific warm-up. Afterwards, the group walked with a moderate pace on varying paths for 70 min using the Nordic walking technique. Specially designed poles were used in this technique to push against the ground with each stride and activate the upper body. The session ended with a five-minute cool-down session identical to that in the climbing group.

#### 2.3.3. Social Contact Control

Participants allocated to the social contact group received brief background information and then watched a movie in the group sessions. Movies included four animated and feature films on anxiety and PTSD-related topics. One full movie was presented within two sessions, followed by a guided group dialog. The group dialog lasted approximately 30 min and focused on a communicative exchange regarding the movie and anxiety/PTSD-related topics.

### 2.4. Study Outcomes and Instruments 

#### 2.4.1. Parameters for Feasibility and Acceptance of the Climbing Program

Feasibility and acceptance of the climbing program was measured throughout the study. To be considered as feasible, the climbing program must result in a reduction in symptom severity of anxiety and PTSD as well as in an improvement in health-related characteristics (e.g., reduced depressive symptoms, lower worry symptoms, growth in self-efficacy, higher quality of life). Acceptance was measured by comparing the number of participants in the climbing group and their follow-up measurements to those of the other group programs and previous findings (e.g., drop-out). Furthermore, the degree of severity of psychiatric disease was recorded to allow comparison with other clinical trials that were conducting exercise interventions with psychiatric patients.

#### 2.4.2. Psychological Parameters

Symptom severity of anxiety disorder was measured by the Beck Anxiety Inventory (BAI) [39]. For patients diagnosed with PTSD, the symptom-specific Post-traumatic Stress Disorder Checklist (PCL-5) [40] was additionally applied. 

Moreover, health-related characteristics for patients with anxiety disorders or PTSD were considered: As symptoms of depression are likely to be present in patients with anxiety disorders or PTSD, depression was measured with the Beck Depression Inventory (BDI-II) [41]. Worry symptoms are a disorder-comprehensive outcome and were assessed with the Penn State Worry Questionnaire (PSWQ) [42,43]. Self-efficacy was measured by the General Self-Efficacy Scale (GSE) [44], an inventory to assess perceived self-efficacy to predict coping with daily hassles as well as adaptation after experiencing stressful life events that might be relevant for clinical practice and behavior change. Quality of life was assessed with the World Health Organization Quality of Life–BREF (WHO QOL-BREF) [45] and includes four subscales for the domains of physical (QoL_phy) and psychological (QoL_psy) health, social relationships (QoL_soc), and environment (QoL_env).

#### 2.4.3. Biological Parameters

Neopterin concentrations were measured by enzyme-linked immunosorbent assay with a sensitivity of 2 nmol/L (BRAHMS Diagnostics, Berlin, Germany). Neurotransmitter precursor amino acids and derivatives (phenylalanine (Phe), tyrosine (Tyr), tryptophan (Trp) and its metabolite kynurenine (Kyn)) were analyzed in the serum by reverse-phase high-performance liquid chromatography, as described elsewhere [46,47]. In brief, sample preparation included protein precipitation by trichloroacetic acid and addition of 3-nitro-L-tyrosine as the internal standard. Phe, Tyr, and Trp were detected based on their natural fluorescence (Trp: excitation and emission wavelengths of 286 and 366 nm; Phe and Tyr: excitation and emission wavelengths of 210 and 302 nm). Kyn and the internal standard 3-nitro-L-tyrosine were detected at a wavelength of 360 nm. The Kyn-to-Trp ratio was calculated to indicate immune-induced Trp breakdown if other inflammation markers such as neopterin were increased simultaneously [48]. The Phe-to-Tyr ratio was calculated as a surrogate of phenylalanine hydroxylase activity [49].

Blood sampling was performed without fasting, between 8 am and 10 am one to three days before the program (t1) and one to three days after the program ended (t2). Therefore, a peripheral venous catheter was inserted into the antecubital vein of the nondominant hand in all participants. Participants rested for 30 minutes following the insertion of the catheter before the initial (resting) blood sample was drawn. The first 2 mL were discarded during each draw. Blood was drawn without stasis. The samples were collected in commercially available tubes (Sarstedt, Vienna, Austria). For analysis of neurotransmitter precursor amino acids aliquots of serum, samples were processed immediately and shock-frozen in liquid nitrogen and stored at −80 °C until use. Furthermore, routine laboratory values were collected to ensure physical health and detect any adverse events that might occur. Routine blood parameters were determined at the hospital standard laboratory.

### 2.5. Statistical Analysis

Data were analyzed using SPSS version 27 (IBM, New York, NY, USA). The statistical analysis was different for psychological and biological parameters. For all psychological parameters including symptoms of anxiety and depression, a pre-planned series of mixed 3 × 4 analyses of variances (ANOVA) was conducted. The ANOVA contained one between-subject factor group (3 levels: climbing group, Nordic walking group, and social contact group) and one within-subject factor time (4 levels: t1 (pre-program), t2 (post program), t3 (follow-up three months post program), and t4 (follow-up six months post program)). The time-by-group interaction analysis was considered the primary analysis of interest and was interpreted as different changes in psychological parameters across groups. The main effects of the factors time and group were also reported. Bonferroni-corrected post hoc tests were conducted as follow-up analyses for the main effects of group or time. Preliminary analysis included tests on homogeneity of variances (Levene test) and on sphericity (Mauchly test). Homogeneity of variances could be assumed in all analyses. Whenever sphericity could not be assumed, the Greenhouse Geisser correction was applied. Partial η^2^ was used as effect size. The analysis of the psychological parameters was done on an intention-to-treat approach. All missing values were imputed using the last observation carried forward method. For all biological parameters, difference variables between t1 and t2 did not show a normal distribution (Shapiro–Wilk test). Therefore, group differences in the change from t1 to t2 were analyzed using the Kruskal–Wallis test. Due to the number of missing values in the biological parameters, we abstained from an intention-to-treat approach for the biological parameters and used a per-protocol analysis. *p*-values below 0.05 were considered as significant (two-tailed). Although a large number of outcomes were analyzed, a type I error adjustment for multiple outcomes was not applied in order to avoid increasing the likelihood of type II errors [50]. Data are presented as the mean (M), standard deviation (SD), and median (Med).

## 3. Results

### 3.1. Description of Study Participants

**Figure 2:**
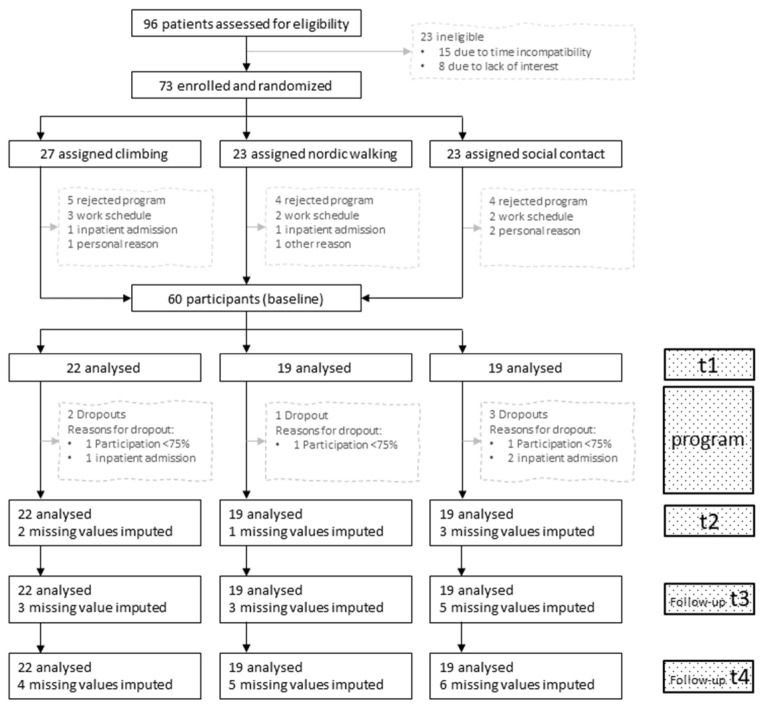
Participant flow. Notes: t1: before program, t2: at the end of the program, t3: three-month follow-up, t4: six-month follow-up.

Between 16 October 2017 and 18 December 2019, 96 patients attended information meetings. Of these, 23 declined to participate due to time incompatibility (n = 15) or lack of interest (n = 8), resulting in a drop-out rate of 24% before randomization. Due to the nature of recruitment, it is not possible to know how many people were aware of the study but did not attend an information meeting. Seventy-three patients were randomly assigned to a group after the information meeting. Of those, 13 patients rejected participating in the program (n = 5 for the climbing group, n = 4 for the Nordic walking group, n = 4 for the social contact group), resulting in a drop-out rate of 18% after random allocation (Figure 2). 

The total sample size was n = 60 patients (mean age 44.2 ± 13.2 years, 75% female). Twenty-two patients participated in the climbing group, 19 in the Nordic walking group, and 19 in the social contact group, excluding two drop-outs for the climbing group, one drop-out for the Nordic walking group, and three drop-outs for the social contact group during the intervention program (Figure 2). Follow-up measurements were conducted between 11 November 2017 and 11 August 2020. Table 1 shows the sociodemographic and health-related characteristics in each of the three groups, according to the medical charts of the Department of Psychiatry, Psychotherapy, Psychosomatics and Medical Psychology Innsbruck only (for the detailed description see Section 2.2). 

Most patients (95%) met the criteria for treatment resistance according to [8,9]. Patients were diagnosed with a mean number of 3.8 psychiatric diagnoses after seeking help in acute care hospitals with ongoing treatment in outpatient care, on average for the past 10.1 years. At study inclusion, three patients (5%) were underweight (body mass index < 18.5 kg/m^2^), 19 patients (32%) were overweight (body mass index > 25–29.9 kg/m^2^), and twelve patients (20%) were classified as adipose (body mass index > 30 kg/m^2^). 

No harmful or adverse event was observed in any of the group programs. In addition, all patients were able to participate in the exercise groups. For example, participation in the exercise groups was observed as feasible with a minimum body mass index of 18 kg/m^2^ and a maximum body mass index of 35.6 kg/m^2^ for a patient in the climbing group and of 17.7 kg/m^2^ minimum and 42 kg/m^2^ maximum for a patient in the Nordic walking group in each case. 

Furthermore, group leaders reported the following challenges and difficulties over the intervention periods: in the climbing programs, two patients could not follow the group program and had to be instructed and supervised in the climbing practices individually due to pain in upper extremities such as fingers and wrist. One patient with a history of sexual assault experienced difficulties with physical touch during belaying, which were addressed and subsequently could be managed by switching from a male to a female rope partner. In the Nordic walking programs, four patients were more physically fit, and three patients were athletically weaker compared to the rest of their group. Therefore, groups were divided into a fast and slow group. Two patients were unable to master the Nordic walking technique and therefore kept walking without poles after the fourth and sixth session, respectively. In the social contact control programs, there were a total of three participants who expressed interpersonal problems with other participants (for example, not wanting to express personal topics in front of the other person) whose participation was hindered as a result. 

### 3.2. Psychological Parameters

No significant time-by-group interactions were found for the psychological parameters, indicating a similar development over time on psychological parameters across the three groups (Table 2). 

**Figure 3:**
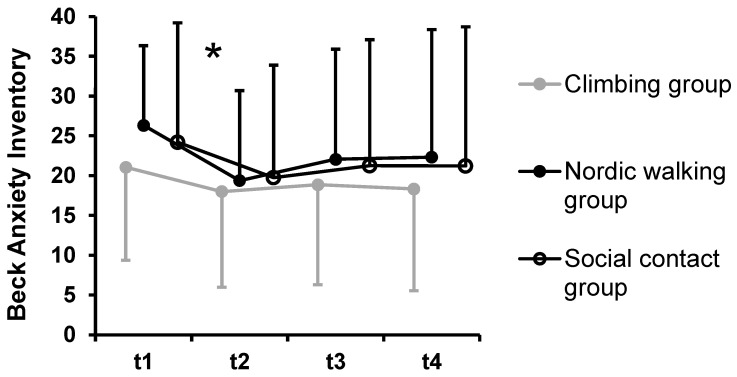
Symptoms of anxiety according to the Beck Anxiety Inventory-II by groups. Notes: * indicates a significant main effect of time. No significant group-by-time interaction emerged. Dots represent mean values; error bars indicate standard deviations.

**Figure 4:**
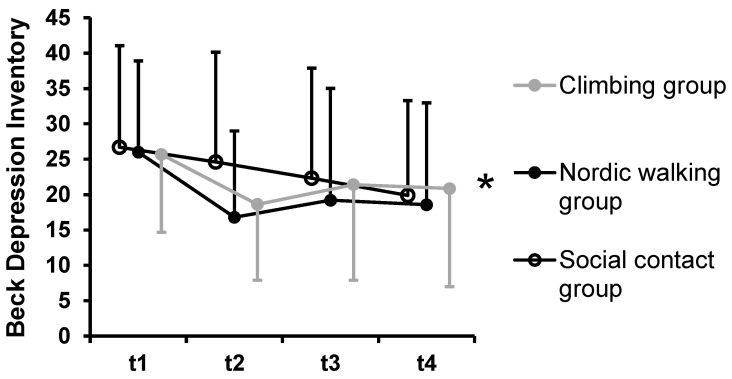
Symptoms of depression according to the Beck Depression Inventory by groups. Notes: * indicates a significant main effect of time. No significant group-by-time interaction emerged. Dots represent mean values; error bars indicate standard deviations.

Significant main effects of time were found for BAI, BDI-II, PSWQ, GSE, and QoL QoL_phy, QoL_psy. Bonferroni-corrected post hoc tests indicated a decrease of symptoms of anxiety from t1 to t2, *p* < 0.001 for the whole sample (n = 60) (Figure 3). The pairwise comparisons to other time points were not significantly different, *p* > 0.107. Symptoms of PTSD decreased from t1 to t2 with a trend to statistical significance. This analysis only included n = 17 patients who were diagnosed with PTSD. Symptoms of depression were significantly higher at t1 compared to those at all other time points, *p* < 0.008 (Figure 4). The pairwise comparisons between t2, t3, and t4 were not significantly different, *p* > 0.999. Similarly, PSWQ decreased over time with the significantly highest value at t1 compared to that at all other time points, *p* < 0.027. The pairwise comparisons between t2, t3, and t4 were not significantly different, *p* > 0.999. 

Self-efficacy and the psychological and physical domains of quality of life showed a significant increase over time. Self-efficacy showed significantly lower scores at t1 compared to those at all other time points, *p* < 0.010. The scores of the psychological and the physical domains of quality of life were significantly lower at t1 compared to those at t2, *p* < 0.013, and significantly lower at t1 compared to those at t4, *p* < 0.003. All other pairwise comparisons were not significantly different, *p* > 0.118. QoL_soc and QoL_env did not show significant main effects of time.

A significant main effect of group was found for QoL environment. The Nordic walking group showed significantly higher values compared to those in the social contact group, *p* = 0.009. Other pairwise comparisons were not significantly different, *p* > 0.309. 

### 3.3. Biological Parameters

No significantly different changes from t1 to t2 across groups were found in any of the biological parameters (Table 3). 

## 4. Discussion

To our knowledge, this was an initial pilot study to assess psychological parameters in combination with biological health-related parameters of two different exercise programs compared to those in a social contact control program in outpatients with anxiety disorders or PTSD. The study has demonstrated the acceptance and feasibility of a climbing program as an add-on treatment for severely affected psychiatric patients with prevalent chronic and treatment-resistant anxiety disorders or PTSD. Since anxiety symptoms and health-related characteristics improved, no adverse events occurred, and drop-out rates for the standard exercise group and the social contact group as well as the average drop-out for exercise trials were comparable, climbing can be considered as an add-on treatment option in this patient population. The study shows that the climbing program, similarly to a Nordic walking and a social contact program, reduced anxiety symptoms. However, it is also possible that the improvements could be due to the accompanying outpatient care. In the present study, no biological parameters to identify underlying pathobiological mechanisms could be identified. Although the present study must be considered cautiously due to the small sample size, the present results do not suggest more beneficial effects of exercise (climbing or Nordic walking) on the change of psychological and biological parameters when social contact is controlled. In contrast to studies about patients with depressive disorders, the climbing program of the present study was not found to have a measurably greater effect than other exercise or social control programs. The results of bouldering psychotherapy in outpatients with depressive disorders provide evidence for superior effects on symptom reduction compared to a waitlist control group [20]. Bouldering psychotherapy compared with a home-based supervised exercise program also results in symptom reduction [19] and enhanced self-efficacy [28]. 

Based on the current scientific literature, there is considerable evidence for the presence of positive exercise effects on individuals with anxiety and PTSD [13,51]. One reason for the absence of an exercise effect in the present study may be based on the patient sample. It might be a question of chronicity, since the mean number of psychiatric diagnoses, years in treatment, number of psychiatric consultations, and patients using prescribed psychopharmacological medication were unusually high in the population participating in this add-on study treatment. For example, around half of the participants in the studies [19] and [28] were using prescribed antidepressant medication compared to 83% of the present sample. Participants of this study were mostly chronic, treatment-resistant patients with anxiety disorders or PTSD with severe mental burden, and this should be considered when comparing the present results with those of other exercise interventions. 

A biological effect of exercise on acute state anxiety has already been reported to be inversely correlated with Kyn/Trp-levels [32]. In the present study on long-term health-related effects, biological data did not confirm the hypothesized changes in Kyn/Trp-levels after participating in an exercise program. In addition, the expected effect of exercise used in the sample size calculation may have been overestimated. This large effect was assumed based on intervention studies, which often used wait lists instead of active control groups. It is known from previous studies that results vary due to different types of control conditions. No effects of exercise were shown when exercise interventions were compared to other conditions like cognitive behavioral therapy, psychoeducation, or meditation [16,22]. Instead, trials using waiting list controls report advantageous exercise effects [16].

It may be the immediate affective responses that should be considered and focused on. Affective responses to certain interventions can be easily controlled in studies and may have long-term effects on mental health. In contrast, symptom severity can be influenced by many other factors such as ongoing psychological or pharmacological treatments. There may also be effects involved that are not adequately measurable with quantitative methods.

Another reason for the lack of expected positive effects could result from the short duration of the exercise programs: The present study implemented a four-week intervention program with two 90-minute sessions per week. In comparison, studies [20,21] included an eight-week program with one three-hour session per week, whereas [19,28] implemented a ten-week program with one two-hour session per week.

Nevertheless, it is undisputed that exercise provides physical health benefits [52]. Especially patients with anxiety disorders and PTSD engage less in physical activity than the general population, further increasing their mortality risk [4]. Offering different exercise therapy programs could potentially provide further benefits for positive affectivity and to the mental state of patients with severe anxiety disorders and PTSD and possibly minimize other health risks as well. 

It is reasonable to conclude that social group effects are of importance. Since patients in the social contact group also benefited from the program, the social factor of group participation seems to be important, possibly even more so than the exercise itself. Thus, group participation per se may counteract the social withdrawal associated with symptoms of anxiety and PTSD [53]. Accordingly, trials using social contact groups to control for exercise effects revealed smaller effects than trials without any intervention at all or waiting list control groups [54]. 

The present study concept could not distinguish the effects of group exercise from the effects of social interaction on serotonin levels. Accordingly, in a previous study on neurophysiological changes, enhanced endorphin releasing effects were only shown in a group exercise intervention, not with exercise alone [55].

Nevertheless, the present results suggest an improvement in most psychological parameters over time, albeit similar across all three group programs. Furthermore, psychological improvements for depressive and worry symptoms were also found on top of reduced symptom severity of anxiety and PTSD. In an exercise-control design, Herring and colleagues also found a reduction of worry symptoms in anxiety patients after participating in an exercise program when compared to those in a non-active control group [56].

Like other studies before [57], current preliminary findings presented positive changes in the quality of life after the end of the program, especially in the dimensions of physical and psychological quality of life. Interestingly, the quality-of-life domain that represents satisfaction with the environment (e.g., “How satisfied are you with the conditions of your living place?”) increased only for patients after participating in the Nordic walking exercise group. This was also the only group exercise that exclusively took place outdoors, in the patients’ immediate environment. It is conceivable that patients may perceive their immediate environment more positively through contact and involvement, as also discussed by [58]. Contrary to the assumption, there were no changes in the dimension of social quality of life. This dimension depicted the level of satisfaction with “personal relationships”, “sex life”, and “support of friends”. Unfortunately, none of these questions could adequately reflect the possible effects of a four-week group program.

Self-efficacy also increased after participation in the program. In contrast to the assumption that exercise interventions and especially climbing or bouldering would increase self-efficacy more (such as in [28]), the present results did not indicate any higher effects of the climbing group or the Nordic walking group.

### Strengths and Limitations

To our knowledge, this is the first pilot study comparing two different exercise programs with a social contact control group to determine the effects in individuals with anxiety disorder or PTSD who experience severe mental burden. Since there is a lack of studies assessing psychological, psychiatric, and biological parameters in combination, this study provides opening insights into potential relationships. However, the most crucial limitation of our study is the small sample size, which led to underpowered results and might be followed by potential misinterpretations. The reasons for the small sample size were the single-center approach, the inclusion of biological parameters, which were associated with an increased amount of blood sampling, and the pandemic situation where sometimes groups had to be stopped due to the local COVID-19 restrictions. In order to attract participants, it was necessary to make the program suitable for everyday use. This was accompanied by further limitations that have to be considered when interpreting the findings: The program period of eight sessions was limited in scope. Furthermore, achieving an identical exercise load in climbing and Nordic walking is difficult due to the different physical demands of the exercise modalities. Thus, climbing exercise could have resulted in a higher exercise load, even though the patients were encouraged to exercise at a subjectively moderate intensity. This aspect should be taken into account when comparing Nordic walking and climbing. Another limitation may be the restricted availability of patient histories and characterization as only patients’ medical charts from the Department of Psychiatry, Psychotherapy, Psychosomatics and Medical Psychology at the Medical University of Innsbruck were available. These gave insight into the medication status, provided information of the diagnosis, years of treatment, and hospital visits for inpatient treatment and outpatient consultations. 

## 5. Conclusions

Climbing as an exercise intervention can be applied as a part of therapy for individuals with treatment-resistant anxiety disorders or PTSD. Exercise is readily available and not associated with negative side effects or the stigma of medications and psychotherapy [59]. Instead, exercise is a low-threshold intervention that maintains and improves physical and psychological health. 

However, exercise programs did not appear to be more effective than social contact programs, since no additional improvements at the psychological symptom level, the worry symptom level, self-efficacy, or quality of life were recorded.

No negative effects were found during application of either climbing or Nordic walking and therefore both can be encouraged to be used in add-on treatment. Climbing therapy represents an alternative exercise modality that may help chronic patients with severe mental burden accept regular exercise even if they are not interested in Nordic walking. 

Climbing therapy expands the repertoire of behavioral therapy interventions to promote physical and psychological health and is a good addition to increase the variety of treatment options offered and achieve the best possible individual fit. Both the findings presented here and the findings in [13] confirm the feasibility and usefulness of exercise in patients with anxiety disorders and PTSD. 

Future research should also address insights of patients’ individual experiences raised by qualitative data.

## Figures and Tables

**Table 1:** Sociodemographic, clinical, and health-related characteristics.

	Climbing Group (n = 22)			Nordic Walking Group (n = 19)			Social Contact Group (n = 19)		
Variables									
Age in years: M (SD) Med	44.8	(13)	44.5	44.7	(13)	47.0	43.7	(14)	45.0
Sex: female n, %	15	68%		16	84%		14	74%	
*State of origin: n, %*									
Austria	15	68%		14	74%		13	68%	
Turkey	3	14%		2	11%		3	16%	
Germany	1	5%		2	11%		3	16%	
Italy	2	9%		0	0%		0	0%	
Other country of origin	1	5%		1	5%		0	0%	
Physical activity hours per week: M ± SD, Med	2.7	(4)	1.5	2.5	(3)	2.0	3.4	(4)	2.5
Body Mass Index: M ± SD, Med	25.2	(5)	23.9	26.2	(7)	23.4	27.4	(4)	27.4
Bloodpressure systolic: M ± SD, Med	131.5	(14)	130.5	136.3	(22)	134.5	125.6	(21)	122.5
Bloodpressure diastolic: M ± SD, Med	84.0	(10)	82.5	85.7	(12)	85.5	81.3	(11)	79.5
Smoking: n, %	9	41%		8	42%		5	26%	
Number of cigarettes per day: M ± SD, Med	11.2	(6)	9.0	17.7	(6)	18.8	17.0	(5)	20.0
*Family status*									
Single/separated/divorced/widowed: n, %	13	59%		14	74%		9	47%	
Married/in a partnership: n, %	9	41%		5	26%		10	53%	
Number of Children: M ± SD, Med	1.3	(2)	0.0	0.7	(1)	0.0	0.8	(1)	1.0
Current occupation, yes: n, %	3	14%		8	42%		0	0%	
*Medication status*									
Total number of prescription drugs: M ± SD, Med	2.7	(1)	2.5	3.4	(2)	3.0	2.8	(1)	2.0
Total number of psychopharmacolocial drugs: M ± SD, Med	1.9	(1)	2.0	1.9	(1)	2.0	2.0	(2)	2.0
Use of psychopharmacolocial drugs, yes: n, %	18	82%		16	84%		16	84%	
Use of antidepressant medication: n, %	19	86%		14	74%		16	84%	
Use of antipsychotic medication: n, %	6	27%		7	37%		5	26%	
Use of benzodiazepines: n, %	1	5%		5	26%		2	11%	
Use of anticonvulsives: n, %	3	14%		5	26%		5	26%	
Use of other psychopharmacolocigal medication: n, %	1	5%		0			0		
*Diagnoses*									
Primary diagnosis of an anxiety disorder: n, %	17	77%		15	79%		11	58%	
Primary diagnosis of a PTSD: n, %	4	18%		3	16%		7	37%	
Primary diagnosis of an anxiety disorder combined with PTSD: n, %	1	5%		1	5%		1	5%	
Number of psychiatric diagnosis/comorbidities: M ± SD, Med	4.4	(3)	4.0	3.7	(2)	3.0	3.2	(2)	3.0
One diagnosis only: n, %	5	23%		3	16%		2	11%	
Number of outpatient emergency consultations: M ± SD, Med	92.4	(180)	41.5	86.5	(105)	56.0	60.9	(54)	39.0
Number of previous psychiatric inpatient treatments: M ± SD, Med	8.4	(16)	3.5	4.8	(6)	2.0	3.9	(4)	2.0
Years of previous psychiatric treatment: M ± SD, Med	11.1	(8)	13.0	9.5	(8)	8.0	9.8	(8)	10.0

Notes: M: mean, SD: standard deviation, Med: median, PTSD: post-traumatic stress disorder, information about the medication status and diagnoses according to records of the Department of Department of Psychiatry, Psychotherapy, Psychosomatics and Medical Psychology only.

**Table 2:** Descriptive and inferential values of the psychological parameters of the group comparisons.

	Descriptives M (SD)						Inferential					
	Climbing Group (n = 22)		Nordic Walking Group (n = 19)		Social Contact Group (n = 19)			*p*-Value			Partial η^2^	
Variables	M	SD	M	SD	M	SD	Group	Time	Inter-Action	Group	Time	Inter-Action
Beck Anxiety Inventory_t1	21.0	(12)	26.3	(10)	24.2	(15)	0.648	**0.003**	0.914	0.02	**0.09**	0.01
Beck Anxiety Inventory_t2	18.0	(12)	19.4	(11)	19.7	(14)						
Beck Anxiety Inventory_t3	18.9	(13)	22.1	(14)	21.3	(16)						
Beck Anxiety Inventory_t4	18.3	(13)	22.3	(16)	21.2	(18)						
Posttraumatic Stress Disorder Checklist_t1	45.2	(13)	49.3	(9)	47.8	(19)	0.962	0.072	0.894	0.01	0.18	0.03
Posttraumatic Stress Disorder Checklist_t2	40.0	24.5	40.8	26.0	41.9	24.1						
Posttraumatic Stress Disorder Checklist_t3	43.2	22.9	39.8	23.9	46.3	20.8						
Posttraumatic Stress Disorder Checklist_t4	37.0	22.0	33.8	19.9	39.1	22.9						
Beck Depression Inventory-II_t1	25.7	(11)	26.0	(13)	26.7	(14)	0.716	**<0.001**	0.251	0.01	**0.16**	0.05
Beck Depression Inventory-II_t2	18.6	(11)	16.8	(12)	24.6	(16)						
Beck Depression Inventory-II_t3	21.4	(14)	19.2	(16)	22.3	(16)						
Beck Depression Inventory-II_t4	20.9	(14)	18.6	(14)	19.9	(13)						
Penn State Worry Questionnaire_t1	56.0	(15)	58.6	(13)	55.8	(14)	0.854	**<0.001**	0.290	0.01	**0.12**	0.04
Penn State Worry Questionnaire_t2	50.1	(16)	53.2	(12)	55.1	(12)						
Penn State Worry Questionnaire_t3	52.5	(17)	50.7	(15)	54.9	(10)						
Penn State Worry Questionnaire_t4	50.3	(17)	51.1	(13)	52.2	(14)						
General Self-Efficacy_t1	22.8	(6)	22.5	(6)	21.5	(7)	0.543	**<0.001**	0.108	0.02	**0.11**	0.06
General Self-Efficacy_t2	24.6	(7)	26.3	(7)	22.1	(7)						
General Self-Efficacy_t3	24.3	(7)	25.0	(6)	22.6	(6)						
General Self-Efficacy_t4	23.8	(6)	24.9	(7)	23.7	(8)						
Quality of Life. physical health_t1	53.5	(19)	48.8	(22)	47.5	(21)	0.510	**0.002**	0.152	0.02	**0.09**	0.05
Quality of Life. physical health_t2	56.8	(19)	58.0	(17)	49.2	(21)						
Quality of Life. physical health_t3	55.7	(17)	60.3	(22)	48.5	(23)						
Quality of Life. physical health_t4	57.7	(17)	57.3	(22)	55.2	(23)						
Quality of Life. psychological health_t1	41.1	(20)	38.7	(17)	40.3	(21)	0.728	**0.006**	0.128	0.01	**0.08**	0.06
Quality of Life. psychological health_t2	45.2	(22)	47.5	(19)	42.0	(21)						
Quality of Life. psychological health_t3	45.2	(23)	49.5	(22)	37.4	(20)						
Quality of Life. psychological health_t4	47.0	(24)	47.3	(21)	44.1	(23)						
Quality of Life. social relationships_t1	57.2	(26)	54.4	(25)	57.1	(26)	0.920	0.226	0.207	0.00	0.03	0.05
Quality of Life. social relationships_t2	62.6	(25)	59.6	(25)	53.6	(23)						
Quality of Life. social relationships_t3	56.0	(24)	57.4	(25)	50.1	(27)						
Quality of Life. social relationships_t4	55.9	(29)	59.7	(24)	60.4	(22)						
Quality of Life. environment_t1	67.8	(15)	69.9	(15)	62.6	(18)	**0.011**	0.186	0.273	**0.15**	0.03	0.04
Quality of Life. environment_t2	68.2	(15)	74.4	(14)	60.4	(18)						
Quality of Life. environment_t3	64.8	(17)	76.9	(16)	57.0	(17)						
Quality of Life. environment_t4	69.6	(14)	77.5	(18)	63.5	(19)						

Notes: M: mean, SD: standard deviation, t1: before program, t2: at the end of the program, t3: three-month follow-up, t4: six-month follow-up; bolded numbers indicate statistically significant values; effect size for partial η^2^ < 0.06 is regarded as small, between 0.06 and 0.14 as medium, and >0.14 as large.

**Table 3:** Group comparison of the changes in biological parameters including descriptive values by group.

	Climbing Group (n = 16)		Nordic Walking Group (n = 14)		Social Contact Group (n = 15)		*p*-Value
Variables	M	SD	M	SD	M	SD	
Kyn/Trp_t1	32.0	(9)	31.7	(11)	33.2	(6)	0.948
Kyn/Trp_t2	32.9	(10)	30.4	(6)	32.4	(6)	
Neopterin_t1	7.2	(3)	8.0	(5)	7.5	(4)	0.364
Neopterin_t2	7.4	(3)	7.2	(3)	8.5	(5)	
Phe/Tyr_t1	1.2	(0)	1.1	(0)	1.1	(0)	0.103
Phe/Tyr_t2	1.2	(0)	1.0	(0)	1.0	(0)	

Notes: M: mean, SD: standard deviation, Kyn/Trp: kynurenine-to-tryptophan ratio [µmol/mmol], Phe/Tyr: phenylalanine-to-tyrosine ratio [mol/mol], neopterin [nmol/L], t1: before program, t2: at the end of the program. The *p*-values refer to a Kruskal–Wallis test on the change scores from t1 to t2.

## Data Availability

The datasets generated and/or analyzed during the current study are not publicly available due to the density of detailed patient data on psychiatric diagnoses, treatment periods, sociodemographic backgrounds, etc., and concerns about not preserving the personal anonymity of our study participants. Upon reasonable request, the dataset containing personal information relevant to the given research questions is available from the corresponding author.

## References

[1] Baxter A.J., Vos T., Scott K.M., Ferrari A.J., Whiteford H.A. (2014). The global burden of anxiety disorders in 2010. Psychol. Med..

[2] Bandelow B., Michaelis S. (2015). Epidemiology of anxiety disorders in the 21st century. Dialogues Clin. Neurosci..

[3] Remes O., Brayne C., van der Linde R., Lafortune L. (2016). A systematic review of reviews on the prevalence of anxiety disorders in adult populations. Brain Behav..

[4] Batelaan N.M., Seldenrijk A., Bot M., van Balkom A.J.L.M., Penninx B.W.J.H. (2016). Anxiety and new onset of cardiovascular disease: Critical review and meta-analysis. Br. J. Psychiatry.

[5] Mendlowicz M.V., Stein M.B. (2000). Quality of life in individuals with anxiety disorders. Am. J. Psychiatry.

[6] Kane J.C., Elafros M.A., Murray S.M., Mitchell E.M.H., Augustinavicius J.L., Causevic S., Baral S.D. (2019). A scoping review of health-related stigma outcomes for high-burden diseases in low- and middle-income countries. BMC Med..

[7] Stubbs B., Vancampfort D., Rosenbaum S., Firth J., Cosco T., Veronese N., Salum G.A., Schuch F.B. (2017). An examination of the anxiolytic effects of exercise for people with anxiety and stress-related disorders: A meta-analysis. Psychiatry Res..

[8] Ansara E.D. (2020). Management of treatment-resistant generalized anxiety disorder. Ment. Health Clin..

[9] Katz C., Stein M., Don J., Seedat S., Saree J., Selek S. (2011). A Review of Interventions for Treatment-Resistant Posttraumatic Stress Disorder. Different Views of Anxiety Disorders.

[10] Kandola A., Vancampfort D., Herring M., Rebar A., Hallgren M., Firth J., Stubbs B. (2018). Moving to Beat Anxiety: Epidemiology and Therapeutic Issues with Physical Activity for Anxiety. Curr. Psychiatry Rep..

[11] Hegberg N.J., Hayes J.P., Hayes S.M. (2019). Exercise Intervention in PTSD: A Narrative Review and Rationale for Implementation. Front. Psychiatry.

[12] Schuch F.B., Stubbs B., Meyer J., Heissel A., Zech P., Vancampfort D., Rosenbaum S., Deenik J., Firth J., Ward P.B. (2019). Physical activity protects from incident anxiety: A meta-analysis of prospective cohort studies. Depress. Anxiety.

[13] Ramos-Sanchez C.P., Schuch F.B., Seedat S., Louw Q.A., Stubbs B., Rosenbaum S., Firth J., van Winkel R., Vancampfort D. (2021). The anxiolytic effects of exercise for people with anxiety and related disorders: An update of the available meta-analytic evidence. Psychiatry Res..

[14] Rosenbaum S., Vancampfort D., Steel Z., Newby J., Ward P.B., Stubbs B. (2015). Physical activity in the treatment of Post-traumatic stress disorder: A systematic review and meta-analysis. Psychiatry Res..

[15] Oppizzi L.M., Umberger R. (2018). The Effect of Physical Activity on PTSD. Issues Ment. Health Nurs..

[16] Bartley C.A., Hay M., Bloch M.H. (2013). Meta-analysis: Aerobic exercise for the treatment of anxiety disorders. Prog. Neuropsychopharmacol. Biol. Psychiatry.

[17] Chalder M., Wiles N.J., Campbell J., Hollinghurst S.P., Searle A., Haase A.M., Taylor A.H., Fox K.R., Baxter H., Davis M. (2012). A pragmatic randomised controlled trial to evaluate the cost-effectiveness of a physical activity intervention as a treatment for depression: The treating depression with physical activity (TREAD) trial. Health Technol. Assess..

[18] Bichler C.S., Niedermeier M., Gufler A., Gálffy M., Sperner-Unterweger B., Kopp M. (2021). A case-control study on physical activity preferences, motives, and barriers in patients with psychiatric conditions. Compr. Psychiatry.

[19] Karg N., Dorscht L., Kornhuber J., Luttenberger K. (2020). Bouldering psychotherapy is more effective in the treatment of depression than physical exercise alone: Results of a multicentre randomised controlled intervention study. BMC Psychiatry.

[20] Stelzer E.-M., Book S., Graessel E., Hofner B., Kornhuber J., Luttenberger K. (2018). Bouldering psychotherapy reduces depressive symptoms even when general physical activity is controlled for: A randomized controlled trial. Heliyon.

[21] Schwarz L., Dorscht L., Book S., Stelzer E.-M., Kornhuber J., Luttenberger K. (2019). Long-term effects of bouldering psychotherapy on depression: Benefits can be maintained across a 12-month follow-up. Heliyon.

[22] Luttenberger K., Karg-Hefner N., Berking M., Kind L., Weiss M., Kornhuber J., Dorscht L. (2021). Bouldering psychotherapy is not inferior to cognitive behavioural therapy in the group treatment of depression: A randomized controlled trial. Br. J. Clin. Psychol..

[23] Leichtfried V., Berghold F., Brugger H., Burtscher M., Domej W., Durrer B., Fischer R., Paal P., Schaffert W., Schobersberger W., Sumann G. (2015). Therapeutisches Klettern—Eine Extremsportart geht neue Wege. Alpin-Und Höhenmedizin.

[24] Frühauf A., Sevecke K., Kopp M. (2019). Ist-Stand der Fachliteratur zu Effekten des therapeutischen Kletterns auf die psychische Gesundheit—Fazit: Viel zu tun. Neuropsychiatrie.

[25] Frühauf A., Niedermeier M., Sevecke K., Haid-Stecher N., Albertini C., Richter K., Schipflinger S., Kopp M. (2020). Affective responses to climbing exercises in children and adolescents during in-patient treatment for mental health disorders a pilot study on acute effects of different exercise interventions. Psychiatry Res..

[26] Bichler C.S., Niedermeier M., Hüfner K., Gálffy M., Sperner-Unterweger B., Kopp M. (2022). Affective Responses to Both Climbing and Nordic Walking Exercise Are Associated With Intermediate-Term Increases in Physical Activity in Patients With Anxiety and Posttraumatic Stress Disorder—A Randomized Longitudinal Controlled Clinical Pilot Trial. Front. Psychiatry.

[27] Luttenberger K., Stelzer E.-M., Först S., Schopper M., Kornhuber J., Book S. (2015). Indoor rock climbing (bouldering) as a new treatment for depression: Study design of a waitlist-controlled randomized group pilot study and the first results. BMC Psychiatry.

[28] Kratzer A., Luttenberger K., Karg-Hefner N., Weiss M., Dorscht L. (2021). Bouldering psychotherapy is effective in enhancing perceived self-efficacy in people with depression: Results from a multicenter randomized controlled trial. BMC Psychol..

[29] Wolf M., Mehl K. (2011). Experiential learning in psychotherapy: Ropes course exposures as an adjunct to inpatient treatment. Clin. Psychol. Psychother..

[30] Vancampfort D., Sánchez C.P.R., Hallgren M., Schuch F., Firth J., Rosenbaum S., van Damme T., Stubbs B. (2021). Dropout from exercise randomized controlled trials among people with anxiety and stress-related disorders: A meta-analysis and meta-regression. J. Affect. Disord..

[31] Asmundson G.J.G., Fetzner M.G., Deboer L.B., Powers M.B., Otto M.W., Smits J.A.J. (2013). Let’s get physical: A contemporary review of the anxiolytic effects of exercise for anxiety and its disorders. Depress. Anxiety.

[32] Hüfner K., Galffy M., Egeter J., Giesinger J.M., Arnhard K., Oberacher H., Gostner J.M., Fuchs D., Sperner-Unterweger B. (2020). Acute and Chronic Mental Stress Both Influence Levels of Neurotransmitter Precursor Amino Acids and Derived Biogenic Amines. Brain Sci..

[33] Gostner J.M., Geisler S., Stonig M., Mair L., Sperner-Unterweger B., Fuchs D. (2020). Tryptophan Metabolism and Related Pathways in Psychoneuroimmunology: The Impact of Nutrition and Lifestyle. Neuropsychobiology.

[34] Kink P., Egger E.M., Lanser L., Klaunzner M., Holzner B., Willenbacher W., Kasseroler M.T., Fuchs D., Weiss G., Kurz K. (2020). Immune Activation and Anemia Are Associated with Decreased Quality of Life in Patients with Solid Tumors. J. Clin. Med..

[35] Sperner-Unterweger B., Kohl C., Fuchs D. (2014). Immune changes and neurotransmitters: Possible interactions in depression?. Prog. Neuropsychopharmacol. Biol. Psychiatry.

[36] Lim A., Harijanto C., Vogrin S., Guillemin G., Duque G. (2021). Does Exercise Influence Kynurenine/Tryptophan Metabolism and Psychological Outcomes in Persons With Age-Related Diseases? A Systematic Review. Int. J. Tryptophan Res..

[37] Cohen J. (2013). Statistical Power Analysis for the Behavioral Sciences.

[38] Borg G. (1998). Borg’s Perceived Exertion and Pain Scales.

[39] Beck A.T., Steer R.A. (1990). Manual for the Beck Anxiety Inventory.

[40] Weathers F.W., Litz B.T., Keane T.M., Palmieri P.A., Marx B.P., Schnurr P.P. (2013). The PTSD Checklist for DSM-5 (PCL-5).

[41] Beck A.T., Steer R.A., Brown G.K. (1996). Manual for the Beck Depression Inventory-II.

[42] Meyer T.J., Miller M.L., Metzger R.L., Borkovec T.D. (1990). Development and validation of the penn state worry questionnaire. Behav. Res. Ther..

[43] Glöckner-Rist A., Rist F. (2014). Deutsche Version des Penn State Worry Questionnaire (PSWQ-d). Zusammenstellung Sozialwissenschaftlicher Items und Skalen (ZIS).

[44] Schwarzer R., Jerusalem M. (1995). Generalized Self-Efficacy Scale: Measures in Health Psychology: A User’s Portfolio. Causal and Control Beliefs.

[45] Group T.W. (1998). The World Health Organization quality of life assessment (WHOQOL): Development and general psychometric properties. Soc. Sci. Med..

[46] Neurauter G., Scholl-Bürgi S., Haara A., Geisler S., Mayersbach P., Schennach H., Fuchs D. (2013). Simultaneous measurement of phenylalanine and tyrosine by high performance liquid chromatography (HPLC) with fluorescence detection. Clin. Biochem..

[47] Widner B., Werner E.R., Schennach H., Wachter H., Fuchs D. (1997). Simultaneous Measurement of Serum Tryptophan and Kynurenine by HPLC. Clin. Chem..

[48] Fuchs D., Möller A.A., Reibnegger G., Stöckle E., Werner E.R., Wachter H. (1990). Decreased serum tryptophan in patients with HIV-1 infection correlates with increased serum neopterin and with neurologic/psychiatric symptoms. J. Acquir. Immune Defic. Syndr..

[49] Capuron L., Schroecksnadel S., Féart C., Aubert A., Higueret D., Barberger-Gateau P., Layé S., Fuchs D. (2011). Chronic low-grade inflammation in elderly persons is associated with altered tryptophan and tyrosine metabolism: Role in neuropsychiatric symptoms. Biol. Psychiatry.

[50] Perneger T.V. (1998). What’s wrong with Bonferroni adjustments. BMJ.

[51] Ashdown-Franks G., Firth J., Carney R., Carvalho A.F., Hallgren M., Koyanagi A., Rosenbaum S., Schuch F.B., Smith L., Solmi M. (2020). Exercise as Medicine for Mental and Substance Use Disorders: A Meta-review of the Benefits for Neuropsychiatric and Cognitive Outcomes. Sports Med..

[52] Pedersen B.K., Saltin B. (2015). Exercise as medicine—Evidence for prescribing exercise as therapy in 26 different chronic diseases. Scand. J. Med. Sci. Sports.

[53] Porcelli S., van der Wee N., van der Werff S., Aghajani M., Glennon J.C., van Heukelum S., Mogavero F., Lobo A., Olivera F.J., Lobo E. (2019). Social brain, social dysfunction and social withdrawal. Neurosci. Biobehav. Rev..

[54] Zschucke E., Gaudlitz K., Ströhle A. (2013). Exercise and Physical Activity in Mental Disorders: Clinical and Experimental Evidence. J. Prev. Med. Public Health.

[55] Cohen E.E.A., Ejsmond-Frey R., Knight N., Dunbar R.I.M. (2010). Rowers’ high: Behavioural synchrony is correlated with elevated pain thresholds. Biol. Lett..

[56] Herring M.P., Jacob M.L., Suveg C., Dishman R.K., O’Connor P.J. (2012). Feasibility of exercise training for the short-term treatment of generalized anxiety disorder: A randomized controlled trial. Psychother. Psychosom..

[57] Alexandratos K., Barnett F., Thomas Y. (2012). The Impact of Exercise on the Mental Health and Quality of Life of People with Severe Mental Illness: A Critical Review. Br. J. Occup. Ther..

[58] Tschentscher M., Niederseer D., Niebauer J. (2013). Health benefits of Nordic walking: A systematic review. Am. J. Prev. Med..

[59] Powers M.B., Asmundson G.J.G., Smits J.A.J. (2015). Exercise for Mood and Anxiety Disorders: The State-of-the Science. Cogn. Behav. Ther..

